# Biochemical characterization of an anti-*Candida* factor produced by *Enterococcus faecalis*

**DOI:** 10.1186/1471-2180-12-132

**Published:** 2012-07-04

**Authors:** Raeesh M Shekh, Utpal Roy

**Affiliations:** 1 Department of Biological Sciences, Birla Institute of Technology and Science (BITS) Pilani KK Birla Goa Campus, NH-17B, Goa, 403726, India

**Keywords:** Antimicrobial peptides, Antimycotic peptides, Anti-*Candida*, AMP, *Enterococcus faecalis*

## Abstract

**Background:**

Because *Candida albicans* is resistant to several antifungal antibiotics, there is a need to identify other less toxic natural products, particularly antimicrobial proteins, peptides or bacteriocin like inhibitory substances. An attempt has been made to purify and characterise an anti-*Candida* compound produced by *Enterococcus faecalis*.

**Results:**

An anti-*Candida* protein (ACP) produced by *E. faecalis* active against 8 *C. albicans* strains was characterised and partially purified. The ACP showed a broad-spectrum activity against multidrug resistant *C. albicans* MTCC 183, MTCC 7315, MTCC 3958, NCIM 3557, NCIM 3471 and DI. It was completely inactivated by treatment with proteinase K and partially by pronase E.

The ACP retained biological stability after heat-treatment at 90°C for 20 min, maintained activity over a pH range 6–10, and remained active after treatment with α-amylase, lipase, organic solvents, and detergents. The antimicrobial activity of the *E. faecalis* strain was found exclusively in the extracellular filtrate produced in the late logarithmic growth phase. The highest activity (1600 AU mL^-1^) against *C. albicans* MTCC 183 was recorded at 48 h of incubation, and activity decreased thereafter. The peptide showed very low haemagglutination and haemolytic activities against human red blood cells. The antimicrobial substance was purified by salt-fractionation and chromatography.

Partially purified ACP had a molecular weight of approximately 43 KDa in Tricine-PAGE analysis. The 12 amino acid N terminal sequence was obtained by Edman degradation. The peptide was de novo sequenced by ESI-MS, and the deduced combined sequence when compared to other bacteriocins and antimicrobial peptide had no significant sequence similarity.

**Conclusions:**

The inhibitory activity of the test strain is due to the synthesis of an antimicrobial protein. To our knowledge, this is the first report on the isolation of a promising non-haemolytic anti-*Candida* protein from *E. faecalis* that might be used to treat candidiasis especially in immunocompromised patients.

## Background

Antimicrobial and antimycotic peptides are small cationic and amphipathic molecules, generally with fewer than 50 amino acids. These ubiquitous peptides have been isolated from prokaryotes and eukaryotes in the plant, bacterial, fungal, and animal kingdoms [1,2]. Nature has strategically placed antimicrobial and antifungal peptides as a first line of defence between the host organism and its surrounding environment, because these peptides are able to inhibit quickly a wide spectrum of infectious microbes without significant toxicity to the host organism. When insects are infected within a short period they secrete an array of cationic peptides to combat the invading organism [3]. Although antimicrobial peptides (AMP) are the primary means of combating organisms in lower forms of life, these peptides have an adjunct role in the immune system of phylogenetically more advanced organisms.

There is a large array of antifungal proteins with different structures. In addition to the well-known glucanases [4], chitinases [5], thaumatin-like proteins [6], defensins [7] and ribosome-inactivating proteins [8], there is a diversity of other antifungal proteins such as lipid transfer proteins [9] and protease inhibitors [10].

Both fungi and humans are eukaryotes and at the molecular level, their cells are similar. This makes it more difficult to find or design drugs that target fungi without affecting human cells. Consequently many antifungal drugs cause side effects. Some of these side effects can be life threatening if the drugs are not used properly. Despite chemical therapies, serious fungal infections remain difficult to treat, and resistance to the available drugs is emerging [11]. Antifungals work by exploiting differences between mammalian and fungal cells to kill the fungal organism without dangerous effects on the host. A common theme with most of these wide-spectrum AMPs is that they lyse the cell membranes of the pathogens without harming the host targets. Despite this non-specific mechanism, many of these peptides do not lyse mammalian membranes at concentrations that can inhibit the pathogen [12].

In the last decades, the incidence of fungal infections by pathogenic *C. albicans* and other related human opportunistic yeast species has increased dramatically due to the rise in the number of immunocompromised patients. Several *Candida* species especially *C. albicans* normally inhabit the oral cavity, respiratory and intestinal tracts, and vaginal cavity of humans and animals. In recent years, there has been a marked increase in the incidence of treatment failures in candidiasis patients receiving long-term antifungal therapy, which has posed a serious problem in its successful use in chemotherapy. *Candida* cells acquire multidrug resistance (MDR) during the course of the treatment [13].

Many bacterial strains, and particularly their enzymes, that perform catalysis efficiently at low temperatures are used in a number of biotechnology applications [14]. *Enterococci*, as part of the natural intestinal flora of humans and animals, are known to play an important role in maintaining microbial balance [15,16]. Many different enterocins have been described from *Enterococcus faecalis* and *E. faecium*. Some of these peptides showed activity against *Escherichia coli*[17] and *Salmonella pullorum*[18].

Since the literature on bacterial antifungal proteins is rather scanty compared with that on bacterial bacteriocins, there is a pressing need to explore and isolate from new sources potential bacteria capable of producing novel AMPs and to characterise them for further applications. In the present study, we report the purification and characterisation of an antifungal protein produced by *E. faecalis*, that shows broad-spectrum activity against the indicator organisms, multidrug resistant *C. albicans* with negligible haemolytic activity.

## Results

### Characterization of species

The promising anti-mycotic strain in the present study was determined to be gram-positive cocci, acid producing, non-motile, catalase and oxidase negative. The strain showed good growth at 6.5% (w/v) NaCl at 14 and 37°C. In addition it was esculin hydrolysis-positive as it fermented mannose which is the characteristic of the genus *Enterococcus*. The producer of the anti-mycotic principle was identified as *Enterococcus faecalis* based on its physiological and biochemical characteristic. Based on the 16S rDNA gene sequence, the strain was identified as *E. faecium*[19]. Further, using the primers EM1A and EM1B [20], an amplicon of approximately 685 base pairs was observed on 1.2% (w/v) agarose gel confirming the strain to be *E. faecium.* However, this strain reduced potassium tellurite and produced black colour colonies, indicating the species *E. faecalis.*

The two wild type isolates (DI and WI) of the pathogenic indicator organism were identified as *C. albicans* based on 18S ribotyping. The sequences of the DI and WI isolates showed closest homology (99%) to the sequences of *C. albicans* M60302.YSASRSUA and AJ005123, respectively.

### Determination of inhibitory spectrum

**Figure 1:**
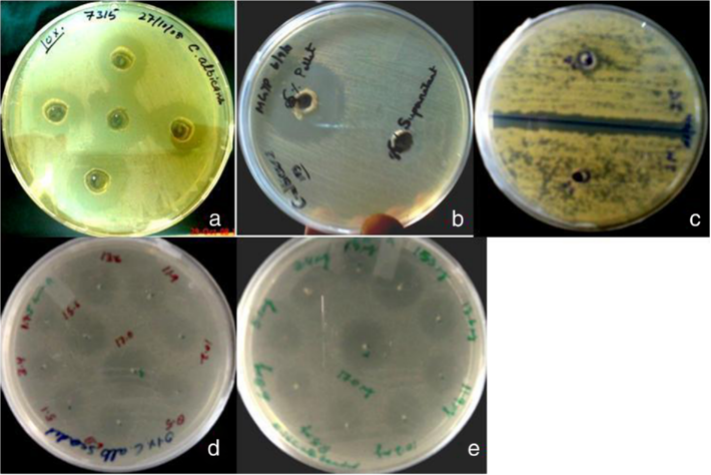
**a. Biological activity of ACP against *C. albicans *(MTCC 7315). b.** Biological activity of ACP against *C. albicans* (MTCC 183) after 85% ammonium sulfate fractionation, The zone of inhibition was detected in 85% palette dissolved in 20 mmol sodium phosphate buffer pH 8.0, but activity was not detected in supernatant. **c.** Mild biological activity of ACP against wild type *C. albicans* (DI) isolated from a diabetic patient in BITS Goa. **d** and **e.** Different concentration of dialyzed concentrate of ACP showing zone of inhibition against a lawn of *C. albicans* MTCC 183.

The susceptibilities of various multidrug resistant *C. albicans* strains to growth inhibition by the supernatant as well as dialysed concentrate of *E. faecalis* are presented in Table 1. The supernatant and dialysed concentrate also showed inhibitory activity against one wild type *C. albicans* strain (DI) isolated from a diabetic patient from Goa. Amongst these strains, maximum activity was observed against *C. albicans strains* MTCC 183, MTCC 3958, MTCC 7315, and NCIM 3471 and minimum activity was observed against wild type *C. albicans* (DI) (Figure 1a, b, c) and *C.krusei* (data not shown). The biological activity of ACP at different dilutions is shown in Figure 1 (d and e) against MTCC 183.

**Table 1:** **Inhibitory spectrum of anti-*Candida *protein ACP against different indicator organisms**

**Strain**	**Identified organisms**	**Indicator organisms**	**Zone of inhibition**
210	*E. faecalis*	*Yersinia intermedia* (AGM 108–5)	25 mm
		*Candida albicans*	>18 mm
		(NCIM 3471, MTCC183, MTCC 7315,	
		MTCC 227 and MTCC 3958)	
	Dialysed Concentrate	MTCC183 and MTCC 7315	55 mm, 47 mm
		Wild type *C. albicans* (DI)	13 mm

### Antimicrobial activity of cell wall and cytoplasmic extracts

The antimicrobial activity of the cell wall and cytoplasmic extracts of *E. faecalis* was determined using a cut-well agar assay on MGYP and BHI plates. No zone of inhibition was produced against *C. albicans* MTCC 3958, *Pseudomonas aeruginosa* MTCC 741 and *Staphylococcus aureus* MTCC 737 by cell wall and cytoplasmic extracts, establishing that the inhibition was mainly due to extracellular substances.

### Kinetics of antifungal protein production

**Figure 2:**
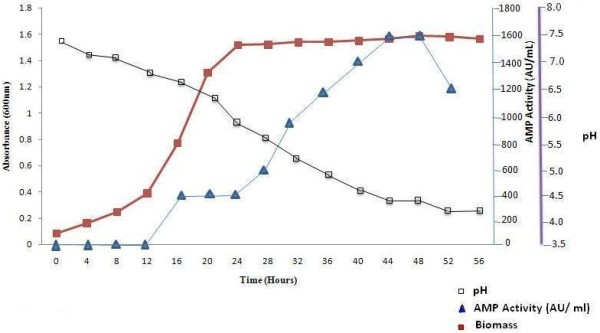
**Kinetics of anti-mycotic protein and biomass production of *E. faecalis.***

Biomass and antimycotic protein production by *E. faecalis* in modified trypticase soya (mTS) broth, was analysed at the incubation temperature of 14°C (Figure 2). This strain reached the stationary phase after 20 h. Prolonged incubation up to 56 h promoted degradation of the ACP but no lysis of biomass. No ACP was produced within 8 h at 14°C, but it was produced during the active growth phase, and its concentration reached a maximum at 48 h, at the middle of the maximum stationary phase. The highest activity (1600 AU mL^-1^) against *C. albicans* (MTCC 183) was recorded between 44–48 h of incubation and decreased thereafter. The pH dropped rapidly during the exponential phase, probably because of the strong production of acid associated with growth.

### Effects of heat, pH, and Hydrolytic Enzymes

The activity of the cell-free supernatant (CFS) was stable upon treatment at different temperatures, for up to 90°C for 20 min, but the activity was lost completely after boiling and autoclaving (Table 2). The antimycotic property of the CFS also remained unaffected at the pH range of 6.0–8.0. However, at pH values of 5.0 and 9.0 the activity was reduced by 50%, whereas at pH values of 2.0, 4.0 and 10.0 the activity was completely lost. The ACP was sensitive to different proteolytic enzymes (proteinase K and pronase E) confirming its proteinaceous nature whereas it was resistant to pepsin, α-amylase, lipase, lysozyme and trypsin at the concentration of 1.0 mg mL^-1^ (Table 2).

**Table 2:** Effect of enzymes, heat, pH, organic solvents and surfactants on the biological activity of ACP (+ve sign, biological activity retained, -ve sign, loss of biological activity)

**Treatment (w/v)**	**Activity**	**Treatment (v/v)**	**Activity**
Trypsin (1.0 mg ml^-1^)	+	Methanol (25%)	+
Pronase E (1.0 mg ml^-1^)	-	Ethanol (25%)	+
Proteinase K (1.0 mg ml^-1^)	--	Iso-propanol (10%)	+
Pepsin (1.0 mg ml^-1^)	+	Hexane (25%)	+
α-Amylase (1.0 mg ml^-1^)	+	Formaldehyde (10%)	+
Lipase (1.0 mg ml^-1^)	+	Chloroform (10%)	+
Lysozyme (2.0 mg ml^-1^)	+	Acetone (10%)	+
37°C, 60°C for 90 min	+	Acetonitrile (70%)	+
90°C for 20 min	+	Triton X-100 (1%v/v)	+
100°C for 30 min	-	Tween-20 (1%v/v)	+
100°C for 90 min	-	SDS (1%w/v)	++
121°C for 15 min	-	Urea (1%w/v)	+
Control at 4°C	+	EDTA (1%w/v)	+
(pH) 6.0, 7.0 and 8.0	+	PMSF (1%v/v)	+
(pH) 2.0, 4.0 and 10.0	-	β-Mercaptoethanol (1 mmol)	+
		DTT (0.1 mol)	+

### Effects of surfactants, organic solvents and storage

The antimycotic peptide ACP remained fully active when treated with different surfactants and organic solvents as mentioned in ‘Methods’. The activity was enhanced by 33.4% in the presence of SDS (1.0%w/v) (Table 2). Long-term storage (1 year) at −80°C did not affect the antimicrobial activity (98%), but a slight reduction (20%) in activity at 4°C and −20°C was found.

### Purification of the anti-*Candida* compound

**Figure 3:**
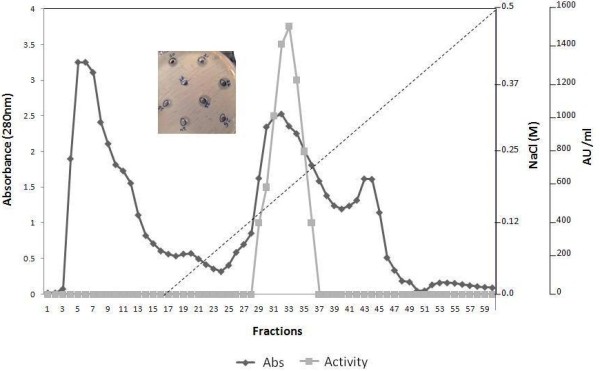
**Chromatogram of antimycotic protein ACP produced by *E. faecalis *on DEAE Sepharose, absorbance of fractions taken at 280 nm.** Fractions (31–35) showing biological activity.

The highest antifungal activity against different *C. albicans* strains was present mainly in the fraction precipitated with 85% ammonium sulfate (Figure 1b). Fractions precipitated with 30% and 50% ammonium sulfate exhibited weak inhibition. The supernatant obtained after 85% ammonium sulfate precipitation clearly did not exhibit any antifungal activity. The antifungal substance present in the 85% cut-off also inhibited germ tube formation in *C albicans* NCIM 3471 (data not shown). As is clear from Table 3, ammonium sulfate precipitation resulted in an approximate 2-fold increase in specific activity. After ion- exchange chromatography using DEAE Sepharose, the adjacent fractions 31–35 in the chromatogram, showed biological activity (Figure 3), and the specific activity increased 17-fold. After gel filtration, the recovery was approximately 22-fold. Based on the purification steps summarised in Table 3, it was concluded that the total active antimycotic protein recovered was 0.45% only.

**Table 3:** Summarised Purification steps of ACP

**Purification stage**	**Volume (mL)**	**Activity (AU mL**^-1^**)**	**Protein (mg mL**^-1^**)**	**Specific activity (AUmg**^-1^**protein)**	**Purification factor**	**Recovery (%)**
Culture Supernatant	400	1600	0.4025	39751	1	100
Ammonium sulfate and dialysis	10	3200	0.0444	72072	1.8	11
Ion Exchange Chromatography	6	1600	0.0023	695652	17.5	0.57
Gel Filtration	2	1600	0.0018	888888	22.4	0.45

### Direct detection of activity on PAGE

**Figure 4:**
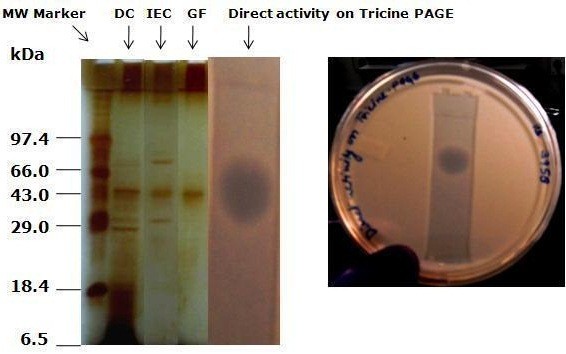
**Tricine-PAGE of ACP purification fractions and gel overlay with *C. albicans *(MTCC 183).** Lane 1, molecular weight marker. Lane 2, dialyzed concentrate after 85% ammonium sulfate fractionation. Lane 3, pooled active fractions collected through DEAE Sepharose matrix. Lane 4, silver stained fractions after gel filtration using Sephadex-G 75. Lane 5, Inhibition zone by antimycotic protein (ACP) on the overlay gel.

After gel filtration, partially purified active pooled fractions (30 μL), were loaded onto Tricine gel containing 10% resolving and 5.0% stacking gel. A clear zone of inhibition on the *C. albicans* MTCC 3958 overlaid gel was shown in a Petri dish (Figure 4), wherein a simultaneously silver stained gel showed a corresponding band that was responsible for the biological activity. Based on the polypeptide molecular weight marker, the molecular mass of the active peptide was estimated to be approximately 43 kDa (Figure 4). We did not observe any biological activity of the bands using glycine Native PAGE.

### Amino acid sequencing

The first 12 amino acid residues of the N-terminal were determined by Edman degradation. The minor sequence obtained from the twice repeated N-terminal sequencing was GPGGPG, and the same partial sequence was matched for homology. Complete homology was not found in the NCBI BLAST result. However, the GPGG sequence matched a known ABC transporter, i.e. ABC Transporter peptide permease and hypothetical protein. The first three amino acid residue GPG matched with N-terminal sequence of enterocin 1071B [21,22]. Likewise the GPG sequence was also observed in EntC2 [23]. Analysis of the major N-terminal sequence DEVYTVKS(S+S’)GLS revealed the presence of S’ suggesting a modified serine which is a feature of class I lantibiotics. This sequence was almost similar to those found in autolysin and hypothetical protein of *E. faecalis*.

### Amino acid composition and sequence analysis done by de novo sequencing

**Figure 5:**
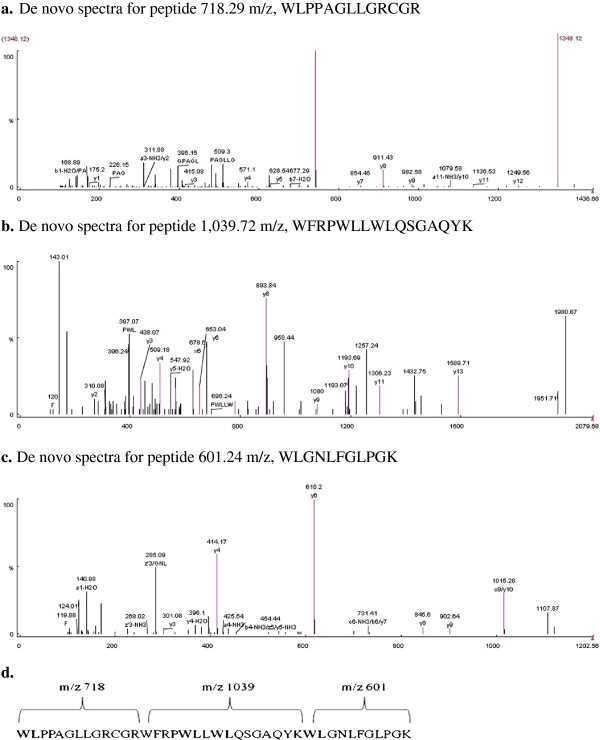
**a. De novo spectra for peptide 718.29 m/z, WLPPAGLLGRCGR. b.** De novo spectra for peptide 1,039.72 m/z, WFRPWLLWLQSGAQYK. **c.** De novo spectra for peptide 601.24 m/z, WLGNLFGLPGK. **d.** Combined de novo sequence of ACP having 3 peptide residues of m/z ratio 718, 1039 and 601.

Based on the de novo sequence the combined peptides having 40 amino acid residues were assembled. Individual peptides having m/z 718, 1039 and 601 were found. The combined peptide did not contain any charged acidic residues (Asp, Glu). Hydrophobic amino acids constituted (42.5%, excluding Gly). The peptides did not significantly match any known proteins present in the MASCOT and BLASTp databases. The amino acid sequence of ACP (40 residues) obtained from peptide fragments after digestion of the antimycotic protein with trypsin was analyzed by MS/MS spectra using PEAKS Studio Version 4.5 SP2 [Bioinformatics Solutions] with subsequent de-novo sequencing. The peaks obtained are indicated in the sequence below, and overlapping residues are shown in bold. The de novo spectra for peptides are given in Figure 5a, b, and c.

Unfiltered BLAST searches using the de novo sequences did not identify any sequence with homology in the Protein Data Bank (PDB). Only a small patch of sequence matched; for example, a WL motif that was found 2 times in enterocin 1071B amino acid sequence [23], and was found 4 times in WLPPAGLLGRCGRWFRPWLLWLQS GAQYKWLGNLFGLPGK in the combined de novo sequence (Figure 5d) of ACP. Earlier study on Ponericin W1 and W2 revealed WL and GL motifs and the presence of hydrophobic residues.

### MIC of the dialysed concentrate containing ACP

**Figure 6:**
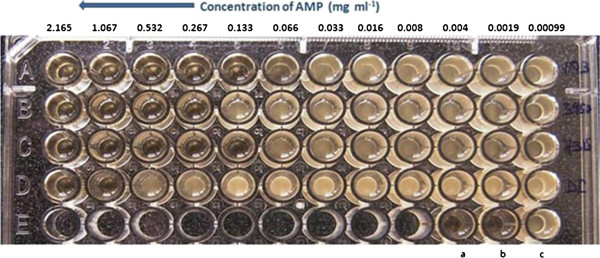
**Antimycotic effect of ACP on the growth of *C. albicans *(MTCC 183, 3958, 7315, and DI), analyzed by a microbroth dilution assay.** Well (**a**) medium only, well (**b**) ACP in the medium only, well (**c**) Grown *C. albicans* in the medium. Rows A–D, normal growth of *Candida albicans,* wells treated with different concentrations of ACP.

The highest minimal inhibitory concentration (MIC), 1067 μg mL^-1^ of dialysed concentrate containing ACP was found against wild type *C. albicans* (DI) whereas the lowest MIC, 133 μg mL^-1^ was found against MTCC 183 and MTCC 7315.The MIC of ACP against MTCC 3958 was 267 μg mL^-1^ (Figure 6).

### Haemolytic and haemagglutination activity assays

**Figure 7:**
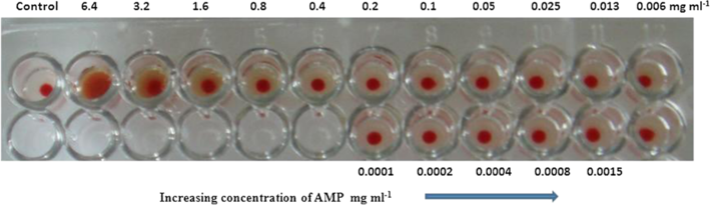
Haemolytic activity of the dialyzed concentrate containing ACP against human erythrocyte cells.

Freshly grown *E. faecalis*, streaked on sheep blood agar plates, did not produce a clear haemolytic zone whereas a clear transparent zone was produced by *Streptococcus pyogenes* and *S. aureus* used as controls. The cytotoxic effect of the extracellular proteins of *E. faecalis* against human RBCs was determined by haemolytic and haemagglutination assays. The effect of various concentrations of the purified anti-*Candida* compound on human erythrocytes is reported in Figure 7. The ACP showed negligible haemolytic activity up to the concentration of 0.4 mg mL^-1^ whereas a very weak haemolytic activity of 3.76% at the concentration of 6.4 mg mL^-1^ of anti-*Candida* protein was found.

**Figure 8:**
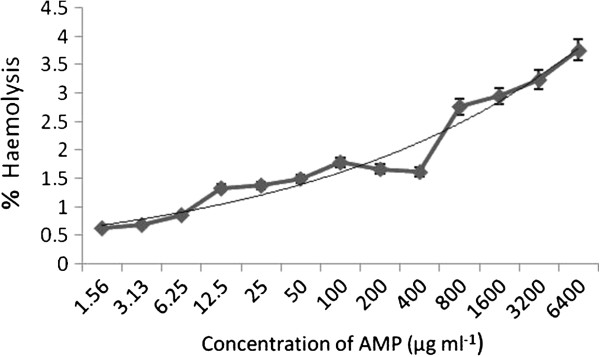
Haemagglutination activity of ACP with different concentration.

No haemagglutination activity of ACP was found up to1.6 mg mL^-1^; however, a slight haemagglutination activity was observed at 3.2 mgmL^-1^ concentration (Figure 8).

## Discussion

 Biochemical characteristics and fatty acid methyl ester (FAME) analysis identified the strain as *E. feacalis*, whereas 16 S rDNA sequencing identified the strain as *E. faecium*[19]. Potassium tellurite reduction, however, distinguished the strain as *E. faecalis* rather than *E. faecium*. The concentrate made from the CFS of the test strain inhibited 7 multidrug resistant strains of *C. albicans.*

 There are several bacteriocins from *E. faecalis* and other species origin [15,24], but antimycotic peptides or proteins are rare. *Pseudomonas syringie* and some *Bacillus species* produce antifungal peptides, but no such reports about *E. faecalis*[25] were found. The genus *Enterococcus* belongs to a group of important lactic acid bacteria (LAB) that participate and contribute towards different fermentation processes. Their functionality in dairy and meat products has been reported in detail [26,27]. Several bacteriocins produced by *Enterococcus* species [24] or other enterococci of different origins [15], have been reported and characterized at the biochemical and genetic levels. Several antifungal peptides (iturins, bacillomycins) were discovered from *Bacillus* and *Pseudomonas.* Nikkomycins, produced by *Streptomyces tendae* and *S. ansochromogenes,* and polyoxins, produced by *S. cacaoi,* are the most widely studied antifungal peptides, whereas antifungal peptides from *Enterococcus* species [25,28] are rare. Various strains of *Bacillus subtilis* produce iturin A and bacillomycin L peptide. Iturins inhibited the growth of fungi including *Aspergillus niger*, *C. albicans*, and *F. oxysporum*[29,30]. Initial clinical trials involving humans and animals showed that iturin A was effective against dermatomycoses and had a wide spectrum of antifungal properties and low allergenic effects [31]. Unfortunately, bacillomycin L and iturin A are haemolytic, which may reduce their potential use as antifungal drugs [32].

 In an era of increased incidence of fungal infections in immunocompromised patients [33,34] and greater resistance to ‘frontline’ antifungal therapies [35], there is a growing need to discover new antifungal therapies. Although newer azole derivatives such as voriconazole are more effective and have cidal activity against filamentous fungi such *Aspergillus fumigatus*[36], these derivatives are fungistatic and not fungicidal against pathogenic yeasts. The inability to kill yeasts leads to resistance to azole in prolonged infections and increases the likelihood that these agents will lack efficacy in severe *Candida* infections in immunosuppressed patients. Amphotericin B has also been commonly used to treat serious fungal infections, but in contrast to azoles, amphotericin B is fungicidal against yeasts. Nevertheless, resistance to amphotericin B is slowly developing in selected *Candida* species [37] and there are significant side effects associated with its use, including nephrotoxicity. Although recently developed antifungal agents, including the peptide-based agents’ micafungin and caspofungin, are very promising, resistance to these therapies has already been reported [38-40] and will no doubt become more widespread. The development of resistance to current antifungal agents, the limited efficacy, and the side effects associated with several of these agents increase the importance of continued development of new alternative approaches.

The identified *Enterococcus faecalis* strain produces the antimycotic substance, ACP, extracellularly. The activity of the ACP was stable upon treatment at different temperatures, for up to 90°C for 20 min but the activity was lost after boiling and autoclaving. While similar results have been reported for bacillomycin D from *B. subtilis*[41] and durancin L28-1A from *E. durans*[42], bacteriocin ST15 from *E. faecium* was inactivated when subjected to 121°C for 20 min [43]. The antimycotic property of the ACP also remained unaffected in the pH range of 6.0–8.0. At pH values of 5.0 and 9.0, however, the activity was reduced by 50% whereas at values of pH 2.0, 4.0, and 10.0 activity was lost completely. These results are similar to those reported for the bacteriocin produced by *E. mundtii*[44]. Several bacteriocins produced by enterococci are known to exhibit a wide range of pH stability [45]. The ACP was stable in different organic solvents and surfactants; such stability has been a common feature of many bacteriocins produced by *Enterococcus*, AMP produced by *Bacillus* species, and other LAB [43,46,47].

 The ACP was fully sensitive to proteinase K and partially sensitive to pronase E, confirming its proteinaceous nature. Its resistance to pepsin, lysozyme and trypsin indicated that the anti-*Candida* active principle may be a cyclic peptide containing unusual amino acids and therefore more resistant to protease hydrolysis [48]. These results suggested that this antimycotic peptide could survive in the intestinal environment and might therefore be administered with food [49]. On the other hand, the ineffectiveness of α-amylase and lipase on antimycotic activity suggested that the ACP might not be glycosylated and might not contain a lipid moiety. When the ACP was heated with 1 mmol and 2 mmol β-mercaptoethaol at 80°C for 10 min to ensure thiol residues existed in the reduced state, no particular change in antimycotic activity was observed. This indicates that the oxidation state of the cysteine residues may not be important for the antimycotic activity [50]. When the dialysed ACP was treated with the reducing agent DTT, no decrease in inhibitory activity was observed, indicating that disulphide bonds are not responsible for biological activity. It was also observed that storage of ACP at −80°C for 1 year did not significantly affect biological activity. Ammonium sulfate salt as well as sodium phosphate buffer did not inhibit ACP activity at the concentration used and did not modify the result of the assay. The dialysed concentrate of ACP, dissolved in 20 mmol sodium phosphate buffer, weakly bound with the DEAE Sepharose matrix, indicating that the ACP bears negative charges. Being weakly negative, it was separated easily in native polyacrylamide gel electrophoresis. After purification by ammonium sulfate fractionation, dialysis, anion exchange chromatography and gel filtration, the final amount of recovered protein (0.45%) was found very low. This could be increased by using protein engineering and optimization methods.

Comparing the partial amino acid sequence of the purified antimycotic protein to other antimicrobial peptides and bacteriocins by using protein-protein BLAST in NCBI revealed no complete homology with other known bacteriocins or AMPs. The combined N-terminal and de novo sequence GPGGPG…WLPPAGLLGRCGRWFRPWLLWLQSGAQYKWLGNLFGLGPK had high amounts of glycine, proline, leucine and tryptophan. This has been observed in many antimicrobial peptides including bacteriocins like enterocin and acidocin.

 It was reported earlier that the glycine-rich antifungal peptide tenacin-3 enters the *C. albicans* cytoplasm [51], although tenacin-3 seems not to induce membrane permeabilisation. Linear peptides with an extended structure were characterised by an unusual proportion of one or more amino acids (most often proline, tryptophan, or glycine) [52,53]. Penaedins characterised from shrimps and prawns had a high content of Pro/Arg/Gly residues in the extended N-terminal domain [54]. Oxypinin 2 has a GVG motif, and ponericin G has glycine residues flanking the central proline, resulting in a GPG motif with calculated grand average of hydropathicity (GRAVY) of −0.683.20. The presence of Gly-Pro hinges in antimicrobial peptides like oxypinins, ponericins, and cecropins supports the antimicrobial potential of ACP, wherein a similar sequence was observed. The regional flexibility provided by proline was sometimes enhanced by the presence of glycine residues [55]. In another recent report, a penaedin homologue, hyastatin from spider crab [56], was shown to possess a Pro/Gly domain similar to the N-terminal domain of penaedins that bind chitin tightly. This information strengthens the idea that the N-terminal minor sequence GPGGPG of the anti-*Candida* protein in the present study could interact with the cell wall of *Candida* as a primer for antimicrobial action [56]. In such a proline-rich sequence, a proline kink has all the potential to create pores [57]. It was cogently argued that in cationic hydrophobic peptides the presence of polar residues confers a hydrophilic property to the proline-rich peptides. In an earlier study conducted on curvaticin FS47, the neutral (Gly [24%]) and hydrophobic (Ala, Ile, Leu, Val, Pro, and Phe [47%]) residues at the N-terminal constitute a significant proportion which helps to explain the hydrophobic interactions that curvaticin FS47 displays. It was reasoned that the high proportion of Gly residues (23.9% in ACP) would likely provide a significant amount of flexibility to the antimicrobial molecule [58]. In fact, the increase of hydrophobicity of the peptides also correlated with fungicidal activity [59]. In accordance with many other bacteriocins of LAB e.g., lactococcin A [60], lactacin F [61], and curvaticin FS47 [58], a high proportion of glycine was likely to provide a significant amount of flexibility to the molecule. A recent study on lactococcin G, enterocin 1071B, and EntC2 suggested that the N-terminal sequence of the peptide of each bacteriocin (LcnGβ, Ent1071B and EntC2) is important for determining target cell specificity [23,62].

Previously, the N- terminal sequence of the antimicrobial dermaseptin B was reported to be highly hydrophobic which could enable its binding to zwitterionic outer and negatively charged surfaces [63]. In addition, the part of the N-terminal sequence which contains Gly-Pro residues and the combined de novo sequence detected in the anti-*Candida* protein ACP 43 under current investigation, were supported by the inference that proline-rich peptides (often associated with arginine) enter cells without membrane lysis and after entering the cytoplasm bind to and inhibit the activity of specific molecular targets causing cell death [64]. Other studies with model amphipathic all L- amino acid peptides with the sequence KX3KWX2KX2K, where X = Gly, Ala, Val, or Leu showed that the leucine-rich peptide, rather than the Ile- or Val-containing peptide, was particularly antimicrobial [63]. Our result is in agreement with this observation: leucine amounted to 19.6%, and proline (13.0%) was in association with arginine.

The combined sequence derived from the de novo sequencing, WLPPAGLLGRCGRWFRPWLLWLQ SGAQY KWLGNLFGLGPK, showed high content of glycine (17.5%), proline, leucine and tryptophan. The amino acid content also revealed that the peptide was quite hydrophobic due to the presence of high amounts of leucine (22.5%), and this is believed to play a role in the interactions with the cell membrane [61]. The hydrophobicities (GRAVY) of individual peptides having m/z 718, 1039 and 601 were 0.108, -0.388 and 0.282 respectively, which indicates that these peptides are relatively hydrophobic and characteristic of many bacteriocins isolated from *Enterococcus* species [65]. High levels of glycine (31%) and glutamine (18%) residues in another cationic antifungal peptide constitutively produced by *S. peregrine* larva were also reported to bind *C. albicans* through electrostatic interaction and disturb the osmotic integrity of treated cells [56]. In contrast, a novel glycine/leucine-rich antimicrobial peptide, leptoglycine (glycine 59.1% and leucine 36.4%) derived from *Leptodactylus pentadactylus* failed to inhibit *C. albicans*. We have used the combined de novo sequence to predict the structure using the PSIPRED (Protein Structure Prediction) server. The sequence WFRPWLLWLQSGAQYK showed alpha helical structure, which is characteristic of many antimicrobial peptides [63].

The MIC of the ACP against wild-type *C. albicans* DI was 1067 μg ml^-1^, whereas the lowest MIC, 133 μg mL^-1^, recorded was against MTCC 183 and MTCC 7315.The MIC of the ACP against MTCC 3958 was 267 μg mL^-1^ which was slightly higher than the MICs of iturin and bafilomycin F [25]. In this study, the results of toxicity experiments were of great interest. ACP was non-toxic to human erythrocytes up to a tested concentration of 6.4 mg mL^-1^. At this concentration, the percent haemolytic activity was 3.76 which is comparatively much less than the haemolytic activities of baciamin [66] and bafilomycin F [25].

It was also concluded that ACP was not able to hemagglutinate human red blood cells up to the concentration of 1.6 mg ml^-1^ (Figure 8), however the concentration higher than this were able to hemagglutinate the human RBC, whereas this concentration is much more than the MIC of the ACP. These properties taken together might render this antimycotic protein ACP, a potent candidate for treating candidiasis, and its related pharmaceutical application can be established in synergy with other relevant antifungal antibiotics of low dosage.

## Conclusions

In this study an antimycotic protein, ACP from the bacterial strain *E. faecalis* was purified to near homogeneity. This antimycotic peptide has negligible haemagglutination and haemolytic activity and hence potentially warrants use in synergy with low dosages of available antifungal drugs to inhibit multidrug resistant *C. albicans.*

## Methods

### Bacterial strains, growth conditions, and media

*E. faecium* (accession number HM481246) was routinely propagated in TGYE medium (tryptone, 5.0 gL^-1^; glucose, 1.0 gL^-1^; yeast extract, 3.0 gL^-1^; pH 7.2-7.4). For ACP production, the strain was grown in optimized mTSB medium (glucose, 2.5 gL^-1^; yeast extract, 2.5 gL^-1^; pancreatic digest of casein, 17.0 gL^-1^; papaic digest of soyabean meal, 3.0 gL^-1^; sodium chloride, 5.0 gL^-1^; K_2_HPO_4_, 2.5 gL^-1^; and pH 7.2). The indicator organism *C. albicans* used in biological activity (cut-well agar) assay was propagated in MGYP (malt extract, 3.0 gL^-1^; glucose, 10 gL^-1^; yeast extract, 3 gL^-1^; peptone, 5.0 gL^-1^, pH 6.4-6.8). The strain was grown in a BOD incubator maintained at 14°C. All microbiological media components were purchased from Hi-Media, Mumbai, India.

Different strains of C*. albicans* were purchased from the Institute of Microbial Type Culture Collection (IMTECH), Chandigarh and National Collection of Industrial Microorganism (NCIM), Pune India. These yeast strains were subcultured regularly in MGYP agar and broth. In the current investigation, the wild-type clinical isolates DI and WI were also used. For their species identification, the fungal genomic DNA was extracted using the kit RTK13. For sequencing the amplicon, ABI 3130 genetic analyser (Chromous Biotech Pvt. Ltd. India) was used.

The test strain was subjected to carbohydrate fermentation using the Hi-Carbo kit KB009-20KT. All strains were stored in appropriate media with 20% glycerol at −80°C.

### Determination of the anti-*Candida* activity

The anti-*Candida* activity was assayed against yeast *C. albicans* MTCC 183, MTCC 3958, MTCC 7315 and NCIM 3471 using the agar-well diffusion assay method as described previously [19]. To determine the titre of the antifungal activity, serial 2-fold dilutions of the extracts were performed. The anti-*Candida* activity was expressed as units AU mL^-1^ corresponding to the reciprocal of the highest dilution causing inhibition of the yeast growth.

### Kinetics determination of *E. faecalis*

The kinetics of antimycotic protein production was determined by inoculating with 1% (10^9^ CFU mL^-1^) of an overnight culture of *E. faecalis* in mTSB enriched broth and incubating at 14°C under uncontrolled pH conditions without agitation. At 4 hours interval, samples were collected to determine the optical density at 600 nm as well as pH. The antimicrobial activity was determined assaying serial two fold dilutions of cell free culture supernatants against *C. albicans* MTCC 183 (10^8^ CFU mL^-1^)*.* The antimicrobial titer was defined in arbitrary units (AU mL^-1^) as the reciprocal of the highest dilution showing inhibition around the well (5.0 mm).

### Preparation of cell wall and cytoplasmic extract

#### Sphaeroplast preparation

*E. faecalis* (4.0%v/v) of was grown in 10 ml mTSB broth at 14°C until the OD at 600 nm was 0.5. The cells were harvested by centrifugation at 10,000 rpm for 10 min at 4°C. The pellet was resuspended at 1/10^th^ the original volume in STE buffer (6.7%w/v sucrose, 50 mmol Tris–HCl 1 mmol EDTA [pH 8.0]) containing 1 mg mL^-1^ lysozyme [67].

The mixture was incubated at 37°C for 30 min and was centrifuged at 5, 00 rpm for 20 min. The supernatant was collected and stored at −80°C until use; the pellet (sphaeroplast) was used to prepare the cytoplasmic extract. The antimicrobial activity of the supernatant was tested against *C. albicans* MTCC 3958, *C. albicans* MTCC 183, *P. aeruginosa* MTCC 741 and *Staphylococcus aureus* MTCC 737.

#### Extraction of cytoplasmic protein

The sphaeroplast obtained was resuspended in hypotonic buffer (50 mmol Tris–HCl, pH-7, 1 mmol MgCl_2_, 25 U RNase A, 50 U DNase 1, [GeneI, India]) [68]. The mixture was incubated on ice for 30 min. Then it was centrifuged at 12,000 rpm for 30 min at 4°C. The supernatant was collected and stored at −80°C until use. The Antimicrobial activity of the supernatant was tested against *C. albicans* MTCC 3958, *P. aeruginosa* MTCC 741, *S. aureus* MTCC 737.

### Physicochemical properties of the anti-*Candida* compound

#### Sensitivity to heat, pH, and hydrolyzing enzymes

Temperature stability was evaluated by incubating the CFS at various temperatures: 60°C for 90 min, 90°C for 20 min, 100°C for 20 and 30 min or autoclaved. Residual anti-*Candida* activity was determined by a well-diffusion assay against *C. albicans*. The effect of pH was determined using a pH range from 2 to 10 adjusted with diluted HCl or NaOH. After incubation at 37°C for 1 h, the resulting CFS was subjected to an agar-well diffusion assay to record the loss or retention of biological activity. Resistance to several proteolytic enzymes was tested by incubating the dialysed concentrate with pepsin, α-amylase, pronase E, trypsin, lipase and proteinase K at a final concentration of 1.0 mg mL^-1^. Buffers were used as controls. Samples were incubated at 37°C for 90 min. The residual activity was determined by cut-well agar assay.

#### Effect of organic solvents, surfactants, and storage

The sensitivity of dialyzed concentrate of ACP was tested in the presence of several organic solvents (methanol, ethanol, isopropanol, hexane, formaldehyde, chloroform, acetone and acetonitrile) at a final concentration of 25% (v/v). After incubation for 2 h at 37°C, the organic solvent was evaporated using a speed vac system (Martin Christ), and the residual antimicrobial activity was determined. An untreated dialysed concentrate sample was taken as control. The effect of various surfactants, including Triton X-100, Tween-20, SDS, urea, EDTA, PMSF, and DTT (1.0% each) on the dialyzed concentrate was also tested. To assess whether the antifungal activity was due to the oxidation state of cysteine residues, β-mercaptoethanol (1 and 2 mmol) was used. The heat-treatment at 80°C was given for 10 min.

In order to determine the stability, the CFS, dialyzed concentrate and partially purified ACP samples were stored for 1 year at low temperatures (4, −20 and −80°C) and the antimicrobial activity was compared to the freshly purified preparation.

#### Partial purification of the anti-Candida compounds

*E. faecalis* was cultured in mTSB medium at 14°C for 48 h. Cells were harvested by centrifugation at 12,000 rpm for 30 min at 4°C, and the CFS was filtered through 0.45 μm membranes. The culture supernatant was subjected to sequential ammonium sulphate precipitation to achieve 30%, 50% and 85% saturation at 4°C with constant and gentle stirring for 1 h. The precipitated proteins were pelleted by centrifugation at 12,000 rpm for 30 min. The protein pellet was dissolved in sterile 20 mmol sodium phosphate buffer pH 8.0, and dialysed using a 10 kDa MWCO membrane (Slide-A-Lyzer Dialysis Cassette, Thermo Scientific) overnight at 4°C, against the same buffer. The crude preparation was then stored at −80°C for further analysis. The 10 mL DEAE Sepharose column (12 cm length and 1.5 cm diameter) was packed. The packed column was equilibrated with 20 mmol sodium phosphate buffer, and 5 mL of dialyzed concentrate was loaded on top of the column. A linear gradient of 0 to 0.25 M NaCl, including 20 mmol sodium phosphate buffer, pH 8, was applied. As many as 60 fractions of 3 mL were collected, and all the fractions were tested for anti-*Candida* activity using the agar-well diffusion assay. The absorbances of all fractions were recorded at 280 nm. All the fractions with antifungal activity were pooled and subjected to ultra filtration (Pall Science) for concentration and removal of salts. Gel filtration chromatography of the pooled active sample was also performed with a Sephadex G 75 column (1.0/45 cm) for final polishing of active protein. The column was eluted isocratically with 20 mmol sodium phosphate buffer, pH 8.0, at a flow rate of 40 mL h^-1^. All the peaks were collected as separate fractions, concentrated by ultra filtration, and tested for antifungal activity using the cut well agar diffusion assay. The absorbance was monitored at 280 nm.

### Direct detection of antifungal activity on gel

Tricine Native-PAGE (10%) [69], followed by a gel overlay was performed with active pooled fractions from gel filtration. After electrophoresis for 2 h at 20 mA, when the dyefront reached at the bottom, 2 duplicate gels were cut. One of the gels was silver stained (based on the Alphalyze protocol). The other gel was fixed in 20% (v/v) isopropanol and 10% (v/v) acetic acid for 30 min, with 500 mL of MilliQ water for 1 h, and placed aseptically on an MGYP plate. To identify the active peptide band, the tricine gel containing pooled active fraction was overlaid by freshly grown *C. albicans* MTCC 3958. After the agar solidified, the plate was incubated at 37°C for 48–72 h until *C. albicans* grew uniformly over the plate or an inhibition zone was observed.

#### Determination of minimal inhibitory concentration (MIC)

The MIC of the dialyzed concentrate against *C. albicans* (MTCC 183, MTCC 3958, MTCC 7315, and wild type *C. albicans* DI from Goa) was determined by the micro- broth dilution assay in a 96-well microtitre plate (Tarsons). *C. albicans* (10^6^ CFU mL^-1^) was tested for sensitivity to 2-fold increasing dilutions of the compounds (2.165 to 0.00099 mg mL^-1^). After incubation at 37°C for 36 h, turbidity was determined to monitor cell growth [70]. The MIC was defined as the lowest concentration of the compounds inhibiting the yeast growth.

#### Haemolytic assay

It was essential first to study the degree of haemolysis produced by the test strain on 5.0% (w/v) sheep red blood cells on blood agar plates. The haemolytic activity of the antifungal dialyzed concentrate on human erythrocytes was determined [71]. Human erythrocytes in 2% (v/v) suspension were exposed to various concentrations of ACP ranging from 6.4 to .00156 mg ml^-1^ at 37°C for 1 h. The cells were peletted at 1,000 rpm for 10 min and the supernatant was collected to determine the absorbance at 450 nm using a UV Visible Spectrophotometer (Shimadzu). In negative control sets, erythrocyte suspension and PBS buffer was used whereas in positive controls, lysis buffer was used for completely lysing the erythrocytes. The percentage haemolysis was calculated and plotted against the concentration of ACP to determine the dose cytotoxic to human erythrocytes. The percentage of intact erythrocytes was calculated using the following formula.

(1) $$Percent\;of\;intact\;erythrocytes=\left(1-\frac{Absorbance\;of\;protein-Absorbance\;of\;PBS}{Absorbance\;of\;lysis\;buffer-Absorbance\;of\;PBS}\right)\times 100$$

(2) Percentofhemolysis=100−(percentofintacterythrocytes)

#### Haemagglutination activity assay

In view of the findings that dialyzed concentrate exhibits haemagglutination activity [72], a serial 2-fold dilution of a solution of ACP (6.4 to 0.0001 mg ml^-1^) was added in microtitre plates, wherein 100 μl was mixed with 100 μl of a 2.0% suspension of human red blood cells in PBS (pH 7.2) at 20°C. The results were observed after about 1 h when the blank without dialyzed concentrate was fully sedimented to inspect whether the red blood cells had agglutinated in response to the antifungal protein.

#### Amino acid sequencing

The corresponding protein band that showed the zone of inhibition against *Candida albicans* was electro blotted to a 0.45 μm Immobilon-P transfer membrane (Millipore). After blotting at 100 mA for overnight, the membrane was removed carefully from the cassette, washed three times with MilliQ water to remove glycine, and then stained for 30 sec with a freshly prepared solution of 0.1% Coomasie brilliant blue R-250 in 40% methanol and 1.0% acetic acid. The blot was then destained in 50% methanol until bands were visible and background clear. The PVDF membrane was then dried sandwiched between clean tissue papers. The stained band of interest was tightly cut out and washed six times in MillQ water and subjected to Edman degradation. The N-terminal sequencing was performed on a Protein sequencer, Model 494 Procise (Applied Biosystems, USA) with 140 C analyzer at Protein Sequencing Facility, IOWA State University, USA. The primary amino acid sequence obtained was entered into BLAST to search for peptides with similar sequences.

#### Mass spectrometry

The purified antimicrobial peptide was analyzed by matrix-assisted laser desorption and ionization–time of flight mass spectrometry by using a 4000 Q TRAP Mass Spectrometer (Proteomics International, Nedlands Australia) equipped with an ion source with visualization optics and an N_2_ laser (337 nm). Protein samples were trypsin digested and peptides extracted according to standard techniques [73]. All digestion reactions were done in 50 mmol NH_4_HCO_3_ (pH 8.5) at room temperature and with an enzyme-to-peptide ratio of 1:40 (wt/wt). Peptides were analyzed by electrospray ionisation mass spectrometry using the Ultimate 3000 nano HPLC system [Dionex] coupled to a 4000 Q TRAP mass spectrometer (Applied Biosystems) with a capillary cap voltage of 1,750 V. Tryptic peptides were loaded onto a C18 PepMap100, 3 μm [LC Packings] and separated with a linear gradient of water/acetonitrile/0.1% formic acid (v/v). MS/MS spectra were analyzed using PEAKS Studio Version 4.5 SP2 [Bioinformatics Solutions]. The mass data collected during LC/MS/MS analysis were processed, converted into mgf files, and compared against the Ludwig NR database by using a local MASCOT server.

The three most abundant peptides, preferably doubly charged ions, corresponding to each MS spectrum were selected for further isolation and fragmentation. The MS/MS scanning was performed in the ultrascan resolution mode at a rate of change in the m/z of 26.000 s^-1^.

## Abbreviations

AMP: Antimicrobial peptide; ACP: Anticandida protein; MTCC: Microbial type culture collection; NCIM: National collection of industrial microorganisms; MDR: Multidrug resistance; DI: Diabetic isolate; WI: Wild type isolate; MGYP: Malt extract glucose, yeast extract, peptone; mTSB: Modified trypticase soya broth; IMTECH: Institute of microbial technology; PMSF: Phenyl-methane-sulfonyl-fluoride; MWCO: Molecular weight cut-off.

## Competing interests

Both authors declare that there is no conflict of interests.

## Authors' contributions

RMS carried out this research (bench work) as part of his PhD work and UR designed several experiments, helped in writing the manuscript and overall supervision of the study. Both authors read and approved the final manuscript.
